# Hygiene heroes: a cluster-randomized trial of a hygiene curriculum in Tamil Nadu schools

**DOI:** 10.1186/s12889-025-25349-6

**Published:** 2025-12-02

**Authors:** David Levine, Ishira Shrivatsa, Malathy Duraisamy, Geetha Karthick

**Affiliations:** 1 Haas School of Business, University of California, Berkeley, CA USA; 2 Department of Economics, University of Chicago, Chicago, IL USA; 3 Department of Humanities, IIT Madras, Chennai, India; 4 Hygiene Heroes and Community Action Trust, Chennai, India

**Keywords:** Handwashing, Behavior change, Cluster-randomized trial, Hygiene intervention

## Abstract

**Background:**

Respiratory infections and diarrheal diseases are major causes of illness and school absences for school-aged children. Both can be prevented by handwashing with soap. Unfortunately, at most schools in low-resource settings, soap and water are rarely present and, if present, rarely used. As part of a longer-term project, we have been working with the Tamil Nadu school system on improving the hygiene practices of students since 2015. We have designed a handwashing curriculum to educate students at low-resource schools in India to promote behavior change among these students and their teachers. The purpose of this study is to measure how effective this curriculum is in improving handwashing outcomes.

**Methods:**

From October 2019 through March 2020, we ran a cluster-randomized trial of a school-based hand hygiene intervention for students in grades 3 to 5 in Tamil Nadu public schools. Schools in the treated group implemented a handwashing curriculum to educate students about the importance of handwashing and to create routines within the school. During baseline, midline and endline, enumerators conducted surprise visits at schools to observe whether soap was present in classrooms and whether students were washing their hands with soap before lunch. The intervention occurred at treatment schools between baseline and midline.

**Results:**

The observed presence of in-use soap and handwashing before lunch more than doubled at treated schools after the intervention. Both outcomes were also roughly 30 percentage points higher at treated schools than at control schools at midline, providing some indication that a hygiene intervention can succeed in a low-resource setting.

**Conclusions:**

Our results indicate that the intervention was successful at improving handwashing with soap. Since the intervention used the school system’s own trainers and teachers, it should be scalable. As such, a hygiene intervention like the one we implemented can succeed in a low-resource setting. Longer-term follow-up is important to see what reminders help schools and students sustain the new behavior.

**Trial registration:**

AEARCTR-0005182 (AEA RCT Registry, Initial Registration Date was December 15, 2019).

**Supplementary Information:**

The online version contains supplementary material available at 10.1186/s12889-025-25349-6.

## Background

Handwashing with soap can reduce the incidence of respiratory infections and diarrheal diseases, which are major causes of school absences for school-aged children [15]. Despite these benefits, at most schools in low-resource settings, soap and water are rarely present, and if present, rarely used [19, 20]. In recognition of this, several studies have implemented handwashing interventions to encourage improved handwashing practices in these settings. Some have focused on providing soap. For instance, Saboori et al. [17] evaluated the effect of soap provision in schools and found positive impacts on handwashing behavior. They noted that results may have been more substantial had there been greater emphasis on a handwashing promotion strategy. Similarly, Bolatova et al. [5] highlight that infrastructure alone is insufficient if factors such as accessibility, maintenance, education, and practices are overlooked. In contrast, other studies have focused on behavioral nudges and have documented greater success [10, 13]. Finally, a further set of studies have combined material provision with structured behavior change efforts, showing improvements in hygiene practices and health outcomes [4, 9, 15]. However, many of these interventions are of high intensity and rely on external staff or intensive short-term campaigns, raising concerns about scalability and long-term sustainability.

These findings underscore the importance of designing handwashing interventions that address behavior change alongside infrastructure, but in a scalable manner. Our study addresses this need by developing and implementing a low-cost, scalable handwashing intervention in primary schools in southern India. Our intervention consisted of two key components. First, we provided schools with soapy bottles (a mix of generic soap and water) and soap refills. This approach offers a more affordable and longer-lasting alternative to bar or liquid soap. Second, we developed and tested a handwashing curriculum that was taught to teachers in schools, so they could implement it as part of their lessons to students. The curriculum is based on a combination of hygiene theories, existing practices, and insights gleaned from past research. To support scalability of our intervention, we trained teachers using the school system’s own trainers rather than external staff.

Our curriculum design is also based on general theories of behavior change. Changing daily behavior is difficult; simply informing individuals of a problem and its solution is rarely sufficient. Familiar examples of challenges include doctors not washing hands between patients [11] and people eating more and exercising less than they intend to [8]. Nevertheless, there are a number of levers to promote behavior change. The Integrated Behavioral Model for Water, Sanitation, and Hygiene (WASH) [7] unifies a number of models (e.g., health behavior change) and levels of influence (e.g. person, situation). Theories of self-efficacy and economic behavior emphasize the importance of ensuring individuals are both capable and equipped to perform the desired behavior [1, 3, 18]. Marketing and behavioral economics focus on the importance of vivid messages and communicating that a behavior is normal in a community [6]. Messages are also more influential if the messenger is a role model whom the listener respects and/or likes. Finally, the literature on habit formation emphasizes the importance of repetition in establishing new behaviors [14].

Drawing on these insights, our curriculum aims to transform handwashing into a routine. Schools in India are adept at implementing daily routines; we attempt to add a simple step: a lead student squirts soap or soapy water on each student’s hands as they leave for lunch, and students scrub on the way to the tap to rinse before eating. Overall, our intervention aims to remove logistical barriers and increase consistent adherence to healthy handwashing behaviors.

## Methods

### Intervention design

Our intervention was designed to promote effective handwashing practices among students at low-resource schools. The focus was on encouraging handwashing at two key times: before eating lunch and after using the toilet. The central idea was that if we could provide schools with inputs and activities to educate students on the importance of handwashing and using soap to eliminate germs, they would begin to incorporate these activities into their normal routines.

Our intervention featured a few key components:

1. We designed a handwashing curriculum that teachers could incorporate into their lesson plans to teach students why, when and how to wash hands with soap.
2. We trained block resource trainer-educators (BRTEs), who are the lowest level trainers and monitors in the government school system in India, to teach teachers how to implement the curriculum and new routines. One of the key practices we emphasized was a routine in which a lead student squirts soapy water onto each classmate’s hands as they walk from the classroom to lunch. The goal of the teacher training was to ensure that teachers understood the health benefits of handwashing for themselves and their students, and to instill a sense of aversion to the idea of invisible germs spreading through their classrooms. We believed that if teachers internalized the importance of handwashing, they would be more effective at conveying its value to their students.
3. We provided soapy bottles and soap refills (Appendix Figure A1). A soapy bottle is a plastic bottle containing a mixture of soap and water. Using soapy bottles, handwashing supplies cost about $0.03 (Rs 3) per student per year.

#### Curriculum design

Our handwashing curriculum featured stories, examples and tutorials, as well as routines to help students learn how to wash their hands. In particular, our curriculum mobilized theories of disgust. Some of the examples we provided involved vivid images of how germs can spread all over a classroom and how both soap and water are required to remove them (Appendix Figure A2). The use of storytelling and images evoking emotion was chosen based on prior success via these methods [4]. Furthermore, a broader set of research has discussed the effectiveness of narrative communication in improving outcomes [12, 16].

To make our curriculum more relatable to students at the target schools, we wrote and illustrated two stories which used existing characters that students would likely be familiar with. The first, “Tenali Rama gets in Trouble”, used the familiar south Indian folk tale character, Tenali Rama. He is a wise man who gives advice to his king. In this story, Tenali Rama arranges for the king to learn that handwashing with soap is essential because filth can be invisible and requires soap to remove. The second short illustrated story uses Chhota Bheem (Appendix Figure A3), a cartoon character who is also familiar to all of the children at these schools.^1^

Since Galiani et al. [9] show that outreach to parents can be effective, our curriculum also featured a letter that students could copy down and take home. The letter asked parents to report whether the student washed their hands at key times: before eating and after using the toilet. We introduced the letters to teach parents what their children (and they) should be doing at these key times, even though they were not a form of data collection that we can apply to our analyses.

To build the habit of washing hands with soap, we included a star chart that teachers could use to record self-reports of washing hands with soap at key times (Appendix Figure A4). The star chart acted as a daily reminder to students. By showing all of students that their peers are washing hands with soap at key times (e.g. before eating lunch and after using the toilet), the star chart also established this pattern as normative in this group.

Finally, to ensure the ability to wash hands effectively, we provided tutorials that teachers could use to demonstrate the proper way to wash hands.^2^

### Intervention implementation

#### Setting

Our handwashing intervention was designed as part of a longer-term project. We have been working with the Tamil Nadu school system (Samagra Shiksha, formerly SSA) on improving the hygiene practices of students since 2015. Our curriculum has been piloted since 2015 in Chennai, India, and then adapted to incorporate feedback and suggestions. To study the effectiveness of our handwashing intervention, we implemented a cluster-randomized control trial (RCT) across 200 schools in the Kanchipuram and Tiruvallur districts in Tamil Nadu. These schools generally comprised of students in grades one through five, with some schools also offering grades six though eight.

The districts included in this study ranged from urban areas adjacent to Chennai to rural and somewhat isolated communities (see Appendix Figure A5). Most students in government schools in Tamil Nadu are from low-income families and many are from Scheduled Castes (formerly called “untouchable” castes). Schools in this region generally tend to have poor learning outcomes. The 2022 Annual Status of Education Report (ASER) shows that in 2018 (prior to the Covid-19 pandemic), approximately 54% of fifth grade students and 25% of eighth grade students in rural government schools in Tamil Nadu could not read a second grade paragraph. In addition, approximately 73% of fifth grade students and 50% of eighth grade students could not do division [2].

#### Randomization and study timeline

To select the schools for the study, we initially chose 10 blocks (a group of schools) in each of the Kanchipuram and Tiruvallur districts so that our data collection area would be more compact. We selected all schools in each block that the Department of School Education reported had at least 80 students, yielding 228 candidate schools. We failed to find six schools and one principal declined to give informed consent, so we collected 221 baseline surveys. After the baseline survey, we dropped one school that had exactly 80 students (our minimum size). We then dropped 20 additional schools, in order of remoteness, to hit our target of 100 schools per district. Finally, schools were randomized into control and treatment groups within these two districts, and treatment schools received the handwashing intervention (curriculum, teacher training, and soapy bottle provision). One of us, who knew nothing about the schools, used Excel’s random number generator to randomly select 50 schools (50%) in each district into the handwashing treatment arm. The other schools received an unrelated dental hygiene intervention and formed the control group in this study.

At each treatment school, the intervention occurred in one classroom for each of the third, fourth and fifth grades. We limited the intervention to primary school students since not all schools offered higher grades. Furthermore, we selected the upper grades in primary school since our intervention works best with increased literacy. If a school had more than one classroom for a grade, we chose the first class in grades three and five and the last class in grade four. Some schools had combined classrooms for grades three and four or for four and five. In such cases, we observed only one or two classrooms in that school. To monitor compliance, we created a WhatsApp group for teachers to send them reminders and to request photos of the implementation of the intervention. Teachers sharing photos on this group reinforced to other teachers that handwashing with soap is a normative behavior for schools in the area.

We implemented three waves of data collection: baseline, midline, and endline. Baseline data was collected from July to October 2019 (prior to any intervention). The teacher training was designed to occur after baseline school observations were completed. The training was conducted separately for each district and took two hours during a regularly scheduled teacher training. BRTEs handed out soapy bottles to teachers during this training. As we discuss in more detail later on, delays in data collection meant that some of our teachers were trained before their school’s baseline survey occurred.

**Fig. 1:**
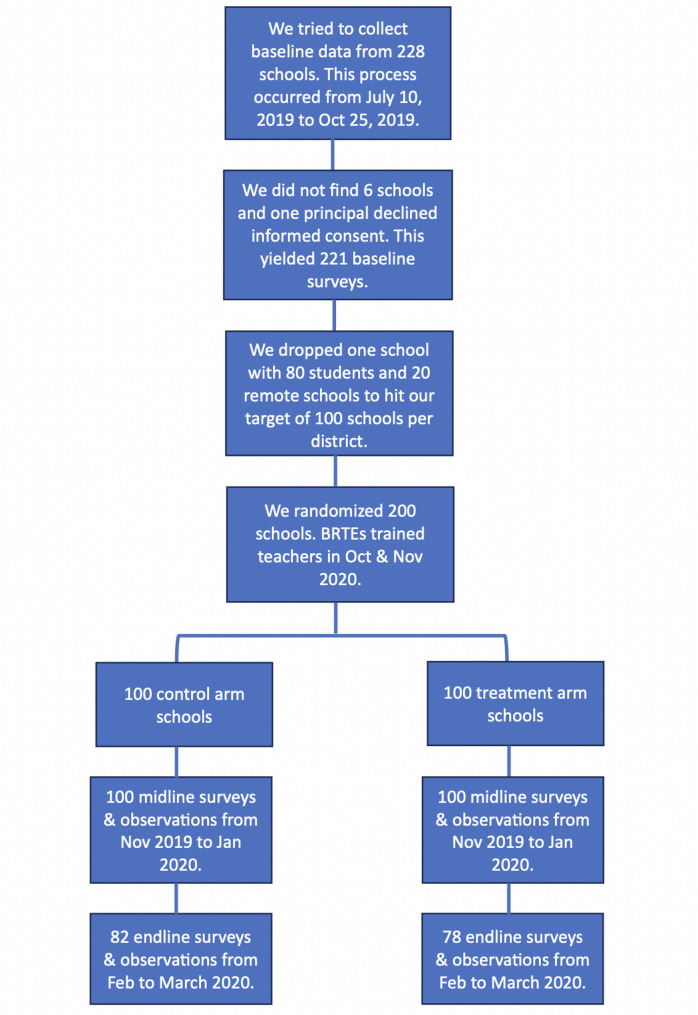
Intervention pipeline Notes: This figure provides detail on the intervention timeline

Midline data was then collected from mid-November 2019 to January 2020, and endline data from February to March 2020. We only completed the endline school observations for 82 control schools and 78 treatment schools because schools in Tamil Nadu closed abruptly due to Covid-19 on March 13, 2020. Figure 1 details the timeline of the data collection process.

### Statistical analysis

Given that we test the effectiveness of our intervention in the form of an RCT, our analysis largely proceeds as a difference in means analysis. Prior to the study, we conducted a power calculation to ensure that our sample size would be large enough to detect meaningful effects. Specifically, we aimed for a sample of 100 treatment and 100 control schools because under reasonable assumptions, we would have 90% power with a one-sided t-test to detect a 15 percentage point increase in handwashing at the 5% level of statistical significance. The assumptions we made include:

1. At baseline, schools with most children handwashing with soap before lunch is equal to 20%.
2. Autocorrelation of school-level behavior is 60%.

#### Outcomes and measurement

While our handwashing intervention was designed to encourage handwashing practices at key times— before eating and after using the toilet— this study focuses on evaluating the impact of the intervention on handwashing before lunch. We have two primary variables of interest. The first is whether soap is present and appears to be in use in up to three observed classrooms (grades three-five) and the second is whether students are observed washing their hands with soap before lunch. While the former indicates the presence of necessary of materials at school, the latter offers a tangible measure of behavior change.

To study the impact of the intervention on the outcomes of interest, enumerators conducted surprise visits at all schools during all three waves of the RCT. The baseline surprise visits were conducted to assess baseline practices at schools, whereas the midline and endline visits were conducted to assess post-intervention outcomes; the enumerators introduced themselves at the first meeting

In terms of measurement, enumerators rated soap as present if soap or a soapy bottle was observed in a classroom. They also noted whether the soap or soapy bottle appeared to be in use as opposed to brand new or empty. For each school, our measure of interest was then the share of classrooms we observed that had soap or a soapy bottle present (and in use) in them. Specifically, an individual school’s observed presence of soap variable was calculated by dividing the number of observed classrooms with an in-use soap/soapy bottle present at that school by the total number of observed classrooms at that school (up to 3 classrooms).

With respect to handwashing before lunch, our primary measure of success was having the majority of students (as judged by our enumerators) wash hands with soap before lunch. In order to observe handwashing before lunch, enumerators conducted their surprise visit a minute or two after the lunch period began. Enumerators defined how many students they observed washing their hands before lunch into four broad categories: “none”, “few”, “most”, and “all”. Due to the limited time enumerators had for observation, they made a rough estimate to define which category their observation fell into. Enumerators were trained to identify each category and did not report any difficulties with these categories during the course of the study. While we acknowledge that a numeric benchmark could have been advantageous, our observation procedure was designed in this way in order to minimize the ability to adjust behavior during observation (Hawthorne effect).

Since our intervention was randomized, effects on both outcomes of interest were analyzed via a difference in means analysis and statistical significance was determined via one-sided t-tests. We include results for within-arm and between-arm differences. We also compute double differences, which we define as follows: for each school, we subtract the outcome at baseline from the outcome at midline (or endline). We then compute an average of this by group, and finally take the difference of the two. Finally, for the handwashing outcome in particular, we are also able to complete some marginal analyses with our data. Since enumerators indicated whether all, most, few, or no students were observed washing their hands before lunch during their visits, we are able to predict the marginal effect of treatment on the probability of being in each of these categories. We conduct this analysis via an ordered logit and control for district and the observed handwashing share at baseline (which could be missing if the school was dropped from the baseline sample). All of our analyses has been completed on Stata.

Going forward, we refer to the observation of soap in classrooms as the “classroom observation” and the observation of handwashing before lunch as the “rapid observation”.

## Results

### Data and balance

We encountered some issues during data collection that reduced the sample size for analysis below what is shown in Figure 1. In some schools, the enumerator’s visit was not a surprise or the enumerator arrived after lunch had begun. The latter issue primarily affected our rapid observation sample since the first few minutes of lunch is the only time when an observation of handwashing before lunch is feasible (classroom observation can still occur as long as the visit is a surprise, but rapid observation could not occur if enumerators were late). For a few schools, delays in data collection meant that teachers were trained before their school’s baseline survey occurred. This reduced both the classroom and rapid observation samples at baseline. Finally, the endline sample (for both types of observation) is further reduced because of school closures due to the Covid-19 pandemic. Table 1 details the number of schools that remain during each wave for each of the two main outcomes.

**Table 1:** Number of schools in final sample

	Baseline		Midline		Endline	
	Control	Treated	Control	Treated	Control	Treated
Classroom Observation	92	82	100	100	79	77
Rapid Observation	74	65	85	92	74	66

*Notes*: This table details our final sample size in each observation dataset

For the classroom observation sample, we began with 100 treated and 100 control schools at baseline. One baseline treated observation was dropped due to incomplete data. We then dropped nine treated and eight control schools because the visits were not a surprise. An additional eight treated schools were dropped because teacher training occurred prior to baseline data collection. At midline, data collection was completed without any issues. At endline, we began with 79 control and 78 treated observations, reflecting incomplete data collection due to COVID-19-related school closures. One additional treated observation was dropped because the visit was not a surprise.

For the rapid observation sample, we also began with 100 treated and 100 control schools at baseline. One observation from each group was dropped due to incomplete data. We then dropped 26 treated and 23 control schools because either the observation was not a surprise or enumerators did not arrive in time to observe handwashing before lunch. Four additional observations (two treated, two control) were dropped due to missing outcome data. Finally, we also dropped six treated schools for which teacher training had occurred before baseline. At midline, we again began with 100 treated and 100 control schools, but dropped 15 control and 8 treated observations due to late arrival of enumerators. At endline, we started with 79 control and 78 treated observations due to COVID-19 school closures. We dropped one treated school because the visit was not a surprise, ten treated and five control schools due to observation delays, and one additional treated observation due to missing outcome data.

To ensure that these changes in our sample did not impact balance, we conducted some balance checks. Table 2 presents a standard balance test comparing treatment and control schools in the classroom observation sample. We failed to reject equality in each variable at any conventional significance level, and also failed to reject a joint test of equality across all variables (P = 0.16). Table 3 replicates these analyses for the rapid observation sample, and shows that we again failed to reject balance (P = 0.32).^3^

**Table 2:** Balance test: classroom observation sample at baseline

	Control	Treated	Diff	*p*-value
Total num students	189.9	184.2	5.8	0.77
Total num classrooms	7.7	7.6	0.1	0.79
Num students in grades 3–5	93.9	100.9	−7.0	0.59
Percent female in grades 3–5	50.2	51.4	−1.2	0.27
Share Kanchipuram	0.5	0.4	0.1	0.39
Num handwash taps	7.8	6.8	0.9	0.23
Number of schools	92	82		

Notes: This table presents baseline characteristics of the schools in the treatment and control groups for the classroom observation sample. The individual *p*-values indicate that the two groups are balanced on various observables. A joint test of significance (via seemingly unrelated regression) across all observables also fails to reject the null hypothesis that the two groups are balanced (*P* = 0.16)

**Table 3:** Balance test: rapid observation sample at baseline

	Control	Treated	Diff	*p*-value
Total num students	192.8	176.2	16.6	0.44
Total num classrooms	7.7	7.3	0.4	0.51
Num students in grades 3–5	97.3	96.2	1.1	0.94
Percent female in grades 3–5	50.4	51.8	−1.4	0.25
Share Kanchipuram	0.5	0.4	0.1	0.31
Num handwash taps	8.2	7.1	1.1	0.23
Number of schools	74	65		

*Notes*: This table presents baseline characteristics of the schools in the treatment and control groups for the rapid observation sample. The individual *p*-values indicate that the two groups are balanced on various observables. A joint test of significance (via seemingly unrelated regression) across all observables also fails to reject the null hypothesis that the two groups are balanced (*P* = 0.32)

### Main results

#### Baseline statistics

During baseline visits, enumerators observed soap in use in about 32% of classrooms at control schools and 26% of classrooms at treatment schools (see Table 4). They also recorded that “most” or “all” students washed their hands with traditional soap or a soapy bottle before lunch at roughly 30% of control schools and 28% of treatment schools (see Table 5). Although the samples used to compute these two measures differ for reasons discussed earlier, we find that among treated schools appearing in both samples at baseline, those with soap present in a larger share of classrooms are, on average, more likely to have “most/all” students observed washing their hands before lunch. For example, among treated schools where only one of three observed classrooms contained soap, 20% had “most/all” students washing their hands before lunch. This proportion rises to 56% among treated schools where all three observed classrooms contained soap.

**Table 4:** Presence of soap and/or soapy bottles– by intervention arm and treatment status

	Baseline			Midline			Endline		
	Control	Treated	Diff	Control	Treated	Diff	Control	Treated	Diff
Avg presence of soap	31.9%	26%	−5.9	58.2%	90%	31.8***	56.3%	87%	30.7***
Avg num classrooms	2.7	2.8		2.6	2.6		2.7	2.8	
Number of schools	92	82		100	100		79	77	

*Notes*: Based on classroom observation sample. An individual school’s observed presence of soap variable is calculated by dividing the number of observed classrooms with soap/soapy bottle present and in-use at that school by the total number of observed classrooms at that school (up to 3 classrooms). *P*-values were calculated via a one-sided t-test. *** *p* < 0.01, ** *p* < 0.05, * *p* < 0.1

**Table 5:** Handwashing before lunch– by intervention arm and treatment status

	Baseline			Midline			Endline		
	Control	Treated	Diff	Control	Treated	Diff	Control	Treated	Diff
Percent of schools with most/all students handwashing before lunch	29.7%	27.7%	−2	32.9%	64.1%	31.2***	40.5%	59.1%	18.6**
Number of schools	74	65		85	92		74	66	

*Notes:* Based on rapid observation sample. *P-*values were calculated via a t-test. *** *p* < 0.01, ** *p* < 0.05, *p < 0.1

#### Presence of soap

Table 4 depicts that the share of classrooms with soap and/or soapy bottles that appeared to be in use more than tripled from 26% at baseline to 90% at midline (P < 0.01) at treated schools. The share also rose from 31.9% to 58.2% for the average control school at midline (P < 0.01). In addition, the difference between treated and control schools in the share of classrooms that had soap which appeared to be in use is highly significant at midline (31.8 percentage points). The endline results are similar, with the average treated school having soap that appears to be in use in 87% of classrooms and the average control school having soap that appears to be in use in 56.3% of classrooms.

Though not reported in a table, we also test the double difference in the presence of in-use soap at midline (for schools with data available at both baseline and midline) and at endline (for schools with data available at both baseline and endline). We define a double difference as follows: for each school, we subtract the outcome at baseline from the outcome at midline (or endline). We then compute an average of this by group, and finally take the difference of the two. The double difference between midline and baseline is 35.3 percentage points (P < 0.01). Similarly, the double difference between endline and baseline is 34.4 percentage points (P $< 0.01$). While the double differences involve a slightly different set of schools than the single differences we report in Table 4, they indicate that our intervention meaningfully increased the presence and usage of soap at treatment schools, despite some potential spillovers to control schools.

It is important to note that the above results do not distinguish between the presence of soap or a soapy bottle. Some classrooms had traditional soap that appeared to be in use, others had only a soapy bottle, and some had both. Appendix Table A1 presents our results by soap type. We find that the large increase in the average presence of soap and appeared usage over the course of the study (at both control and treatment schools) is due to the increased presence of soapy bottles. This is in line with what we would expect, given that we provided treatment schools with soapy bottles.

#### Handwashing before lunch

Table 5 depicts that among the treated group, the share of schools with a majority of students washing their hands with soap more than doubled from 27.7% at baseline to 64.1% at midline (P < 0.01). The observed handwashing at lunch remained about the same for control schools between baseline and midline. At endline, the shares converged slightly to 59.1% for treatments and 40.5% for controls (P < 0.05).

We once again compute double differences at midline and at endline (not presented in Table 5). The double difference between midline and baseline is 40.7 percentage points (P < 0.01). The double difference between endline and baseline is 14.1 percentage points, though this result is not statistically significant. Once again, although the double differences involve a slightly different set of schools, the magnitude of the midline double difference implies our intervention increased proper handwashing before lunch – though the longer-term effect is imprecise.

Finally, Table 6 depicts the average marginal effect of treatment on the probability of being in each category of the handwashing spectrum. Column 1 depicts that at midline, being in the treated group increases the probability of observing most students washing their hands with soap by 29 percentage points (a nearly 100% increase from the baseline control mean) and the probability of observing all students washing their hands with soap by 6 percentage points (holding control variables at their mean values). Similarly, being in the treated group decreases the probability of observing no students washing their hands with soap by 18 percentage points. While these results persist at endline (see Column 2), they are smaller in magnitude and of less statistical significance. A chi square test rejects (at the 95% level) the null hypothesis that treatment does not impact the handwashing with soap outcome at both midline (P < 0.01) and endline (P < 0.05).

**Table 6:** Handwashing before lunch, ordered logit probabilities

	(1)	(2)
	Midline	Endline
none	−0.184***	−0.0664*
	(0.0436)	(0.0307)
few	−0.162***	−0.129*
	(0.0423)	(0.0560)
most	0.285***	0.165*
	(0.0598)	(0.0690)
all	0.0606**	0.0305
	(0.0210)	(0.0170)
$N$	176	139

*Notes:* The coefficients in this table represent the average marginal effect of treatment on the probability of being in each category of the dependent variable (handwashing with soap), holding control variables at their mean values. Control variables include the observed handwashing share at baseline and an indicator for whether or not the school was observed at baseline. Standard errors in parentheses. *** *p* < 0.01, ** *p* < 0.05, * *p* < 0.1

Overall, our analysis suggests that our intervention increased the presence of soap and proper handwashing behavior at schools. Because the final sample of schools in our analysis changes across waves of the RCT, we repeat our analysis in this section using data consisting of only the schools that had valid data at baseline, midline, and endline. The results from this analysis can be found in Appendix A.3 (Appendix Tables A2 and A3 display balance tests, while A4 and A5 display results). We find that the results for both outcomes of interest are comparable to those presented in our main analysis.

### Additional results

Although not part of our analyses, we briefly describe what we learned from the photographs that teachers sent us via WhatsApp. All 100 initial treatment schools sent at least one image, with a mean of 4 photos per school. Slightly over half sent a photo of a star chart, 36% sent a photo of a letter home, and 34% sent a photo of a soapy bottle. Almost all schools also sent various other photos, including those of children washing hands. Having a photo from a school is not evidence that the two or three treated classrooms in that school taught 100% of the curriculum. At the same time, the photos provide some reassurance that every treated school had at least one classroom that worked on at least part of the curriculum.

Some schools showed additional initiative as well. Four schools (4%) posted photos to the WhatsApp group of posters students had drawn and/or a play or a rally on handwashing themes. One school grouped the students by class and gave a trophy to the group that washed hands most consistently each week. Another school had their students prepare an herbal hand wash in the science lab. When one of us went in for the field visit, they demonstrated the soap making experiment.

## Discussion

Our intervention was a randomized evaluation and despite any attrition related issues, our control and treatment groups appear to be balanced on observables. We find that our handwashing intervention was successful. In particular, the presence of in-use soap and handwashing before lunch more than doubled at treated schools after the intervention. Both outcomes were also roughly 30 percentage points higher at treated schools than at control schools at midline.

Before discussing our results in relation to prior literature, we note that our handwashing outcome is constructed differently from many previous studies, which typically measured the proportion of key moments at which handwashing occurred. Therefore, comparisons between our findings and the existing literature should be interpreted with caution. That said, if we directly compare our handwashing results to those from prior interventions, the magnitude of the changes we observe during our intervention is substantial. In particular, we find a 36 percentage point increase (from 28% to 64%) in observed handwashing before lunch between baseline and midline at treated schools. This effect remains sizable at endline, with a 31 percentage point increase relative to baseline. These gains exceed the approximately 25 percentage point increase (from 7% to 32%) reported in Saboori et al. [17], which focused primarily on soap provision. The larger improvement in our setting may reflect the added influence of our behavior change component. Our findings are also comparable to those from the high-intensity SuperAmma RCT in rural India, where handwashing with soap at key times increased from 1% at baseline to 37% after six months [4]. That study also documented a 31 percentage point difference in handwashing behavior between treatment and control groups at follow-up, which is comparable to what we observe at midline. While our intervention shared key elements with SuperAmma, such as storytelling and vivid imagery, it was implemented at much lower intensity and cost. The similarity in outcomes suggests that even low-cost interventions may be capable of producing meaningful behavior change. That said, the results in Biran et al. [4] were measured over a longer time frame than in our study, and longer-term follow-up in our setting is necessary to assess the persistence of these effects.

Our baseline handwashing figure in Table 5 is much higher than figures reported in low-resource areas in other studies. For example, in Biran et al. [4], the baseline level of observed handwashing at key times was 1%. Though the settings and measurement techniques differ, we note that in our team’s observations prior to the RCT, soap was rarely observed in government schools in Tamil Nadu as well. As such, the baseline handwashing figure is likely higher than at most schools in Tamil Nadu. There are a few possible reasons for this result. First, in one district, a local company had distributed soap several months prior to the start of our intervention; this distribution was unrelated to our intervention. In addition, the schools that delayed entry for our enumerator at baseline (so we were unable to observe the baseline soap use before lunch) may have been less likely to have soap. Finally, for baseline observations, we had to call local school officials to find out school locations. These officials sometimes warned principals that we were coming the next day to discuss handwashing.

We now turn to addressing some of the limitations of this study. First, we are unable to separate potential spillover effects from our main results. During follow-up interviews, we learned that some principals and teachers at treatment schools discussed the handwashing intervention with peers at other schools. In addition, BRTEs may not have followed the given randomization and may have taught teachers at control schools about soapy bottles. These occurrences likely led to spillover effects, which can in fact be observed in some of our results itself. We find that control schools experienced in an increased presence of in-use soapy bottles and handwashing with soap from baseline to midline, though by a much smaller magnitude than treatment schools. Finally, although we took measures (e.g. surprise visits) to minimize the Hawthorne effect, enumerators categorically looked for the presence of soap during baseline visits, which may have promoted principles and/or teachers to acquire soap going forward. Given these potential spillover effects, our analysis and results should be interpreted as part of an intention to treat framework.

Another limitation is that the hygiene observation was not blind, which may have led to unconscious bias. Furthermore, we lack specific data on intervention fidelity. There may have been variable implementation of different elements of the intervention between classrooms. Finally, we did not separately test the impact of each component of the intervention or each theory supporting that component. It would be fruitful to break down the intervention and find the most cost-effective subset.

Since the implementation of this RCT, the head of the school system in Tamil Nadu (the joint director of Samagra Shiksha, formerly SSA) has verbally approved scaling our intervention. After the Covid-19 pandemic, the state shifted from training teachers in person to virtual training via videos. In response, we plan to adapt our intervention for delivery via video. As of May 2025, we are seeking approval for the training scripts to proceed with filming short videos and remain hopeful about scaling the intervention to all 37,000 government schools across Tamil Nadu.

## Conclusion

We implemented a theory-based handwashing intervention that emphasized building a low-cost routine involving handwashing with soap. Our results indicate that the intervention was successful at improving handwashing with soap. The success of this intervention depicts the challenges and opportunities of integrating good hygiene into Tamil Nadu schools. For many years, at each World Handwashing Day, Tamil Nadu schools taught students how to wash their hands with soap. Despite this, rates of handwashing with soap remained very low. In contrast, when we both provided soap and targeted behavior change by introducing story-telling, new routines, and incentives (star charts), we saw substantial increases in handwashing with soap at a key time. Since this intervention used the school system’s regular trainers to train the teachers who then delivered content to students, a handwashing intervention like this one can succeed in low-resource settings and can be introduced to scale.

The onset of the Covid-19 pandemic hindered our ability to conduct follow-up observations over time, which made it difficult to assess if and by how much the magnitude of our results would decline over time. Future research exploring longer-term follow-up is important to understand what reminders help schools and students sustain new behaviors, and how the intervention may impact attendance and health outcomes.

## Supplementary Information

Supplementary Material 1.

## Data Availability

The datasets generated and/or analysed during the current study will be accessible via Levine’s website (https://faculty.haas.berkeley.edu/levine/).

## Footnotes

1 We appreciate permission from Emerson College and Green-Gold studios for use of Green-Gold’s characters.

2 More detail and materials related to our curriculum can be found at https://hygieneheroes.berkeley.edu/handwashing.html.

3 Since the number of participating schools in both control and treatment groups varies slightly across waves of the RCT (baseline, midline, and endline), one may additionally be concerned about selection into participation after baseline. To examine this possibility, we checked whether participating schools are balanced across all treatment groups and waves of the RCT. We failed to reject a joint test of equality in the variables considered in Tables 2 and 3 across all treatment groups and RCT waves.
