# Supplementary material for: Hygiene heroes: a cluster-randomized trial of a hygiene curriculum in Tamil Nadu schools
Source: BMC Public Health. 2025 Dec 2;26:85. doi: 10.1186/s12889-025-25349-6 (PMC12777146; doi:10.1186/s12889-025-25349-6)
Supplement: Supplementary file 1 — Supplementary Material 1. [file 12889_2025_25349_MOESM1_ESM.zip › Rapid Observation Survey.pdf]

Rapid Observation

QUESTIONS RESPONSES 2

Rapid Observation

Form description

1.Date of visit for observation

Month, day, year

2. School Name

Short answer text

3. Time start of visit

Time

4. Observer Name

- Antony
- Lakshmanan
- [illegible]

Surendhar

- Geetha
- Others

5. Has this site been trained before

- Yes
- No

If yes, date of training

Month, day, year

6. Reason for visit

- Training
- Observation
- Baseline
- Midline
- Endline
- Others

7. Was it a surprise visit?

- Yes

- Maybe

8. How visible is open defecation?

- Lots
- More
- Some
- None

9. Garbage visible

- Lots
- More
- Some
- None

10.Number of taps

Short answer text

11.Number of operational hand wash station

Short answer text

Short answer text

13.How many latrines/toilets for girls

Short answer text

14. How many staff bathrooms?

Short answer text

15. How dirty are the bathrooms?

- Look and smell ok
- Look and smell pretty good
- Smells disgusting
- Other...

16. Near the toilets do you see soap?

- Yes
- No
- Maybe

17. Are there soap refills at school

No

May be

Other...

18.Chance to observe students before lunch/snacks

Yes

No

If Yes,

Did not wash hands

Washed with water only

Washed with soap

Others

19.Chance to observe students after toilet

Yes

No

If Yes,

Did not wash

Washed with water only

- ○ Others

20. Is drinking water treated?

- ○ Yes
- ○ No
- ○ Maybe

If Yes, What type of treatment,

- ☐ RO
- ☐ Can water
- ☐ Chlorinised water
- ☐ Not Applicable
- ☐ Don't know
- ☐ Other...

21. What is the storage method

- ☐ Container with tap
- ☐ Narrow mouth (have to pour into or have a tap)
- ☐ Wide mouth (wide enough to fit a dipper into it)
- ☐ Other...

22. Is there a tumbler or mug?

- Yes
- No
- Maybe
