# Supplementary material for: Hygiene heroes: a cluster-randomized trial of a hygiene curriculum in Tamil Nadu schools
Source: BMC Public Health. 2025 Dec 2;26:85. doi: 10.1186/s12889-025-25349-6 (PMC12777146; doi:10.1186/s12889-025-25349-6)
Supplement: Supplementary file 1 — Supplementary Material 1. [file 12889_2025_25349_MOESM1_ESM.zip › Follow up - Class Observation Survey.pdf]

Follow up - Class Observation

QUESTIONS RESPONSES

Classroom Observation

Form description

1. Date of visit for observation

Month, day, year

2. School Name

Short answer text

3. Time start of visit

Time

4. Observer names

- Antonyraj
- Lakshmanan
- [illegible]

Sureender

◯ Geetha

◯ Other...

5. Reason for visit

◯ Baseline

◯ Midline

◯ Endline

◯ Observation

◯ Teaching

◯ Other...

6. Was the visit a surprise?

◯ Yes

◯ No

◯ May be

7. Number of classrooms observed

Short answer text

9. Are there soap or soapy bottle in the classroom?

- Yes, and looks unused
- Yes, Could not tell about usage
- No
- Other...

11. Teacher present in Classroom?

- Yes
- No

12. Handwash Arm - Evidence of curriculum taught

- Letter home returned to teacher
- Art on walls
- Empty star charts
- Filled in star charts
- Other...

13. Dental Arm - Evidence of curriculum taught

- Toothbrushes present
- Toothpaste present
- Other...

12. Time end of visit

Time
