# Supplementary material for: Hygiene heroes: a cluster-randomized trial of a hygiene curriculum in Tamil Nadu schools
Source: BMC Public Health. 2025 Dec 2;26:85. doi: 10.1186/s12889-025-25349-6 (PMC12777146; doi:10.1186/s12889-025-25349-6)
Supplement: Supplementary file 1 — Supplementary Material 1. [file 12889_2025_25349_MOESM1_ESM.zip › Baseline - Class Observation Survey.pdf]

QUESTIONS RESPONSES 3

Classroom Observation

Form description

1. Date of visit for observation

Month, day, year

2. School Name

Short answer text

3. Time start of visit

Time

4. Observer names

- Antonyraj
- Lakshmanan
- Chithra
- Sureender

- Other...

5. Reason for visit

- Baseline
- Midline
- Endline
- Observation
- Teaching
- Other...

6. Was the visit a surprise?

- Yes
- No
- May be

7. Number of classrooms observed

Short answer text

8. Number of classes with teacher

- Option 1

9. Are there soap or soapy bottle in the classroom?

Multiple choice

- Yes, and looks used
- Yes, and looks unused
- Yes, Could not tell about usage
- No
- Other...
- Add option

Required

11. Teacher present in Classroom?

- Yes
- No

12. Time end of visit

Time
