# Supplementary material for: Hygiene heroes: a cluster-randomized trial of a hygiene curriculum in Tamil Nadu schools
Source: BMC Public Health. 2025 Dec 2;26:85. doi: 10.1186/s12889-025-25349-6 (PMC12777146; doi:10.1186/s12889-025-25349-6)
Supplement: Supplementary file 1 — Supplementary Material 1. [file 12889_2025_25349_MOESM1_ESM.zip › appendix.pdf]

A Appendix

A.1 Figures

Figure A1: The soapy bottle

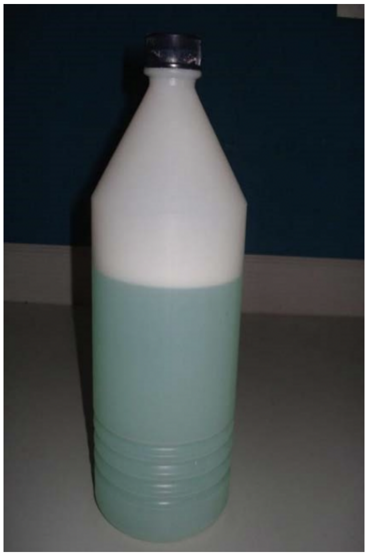

Figure A2: Curriculum creates disgust at hands not washed with soap

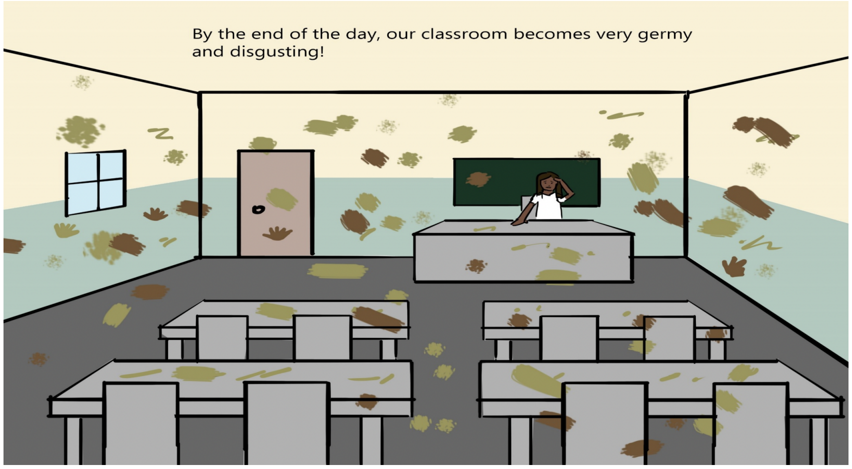

Figure A3: Illustrated story with Chhota Bheem

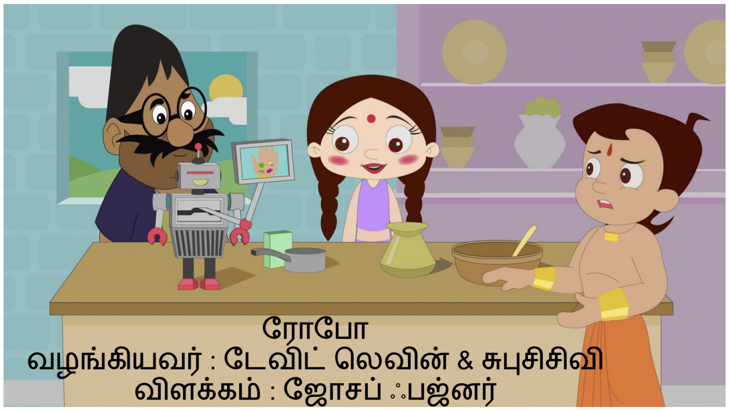

Figure A4: Star chart

Group	Heroes	2	3	4
Monday: Start				
Tuesday: Soapy bottle before lunch				
Wed.: Soapy bottle before lunch & at toilet				
Thursday: Soapy bottle before lunch & at toilet				
Friday: Soapy bottle before lunch & at toilet				

Figure A5: District map of northern Tamil Nadu

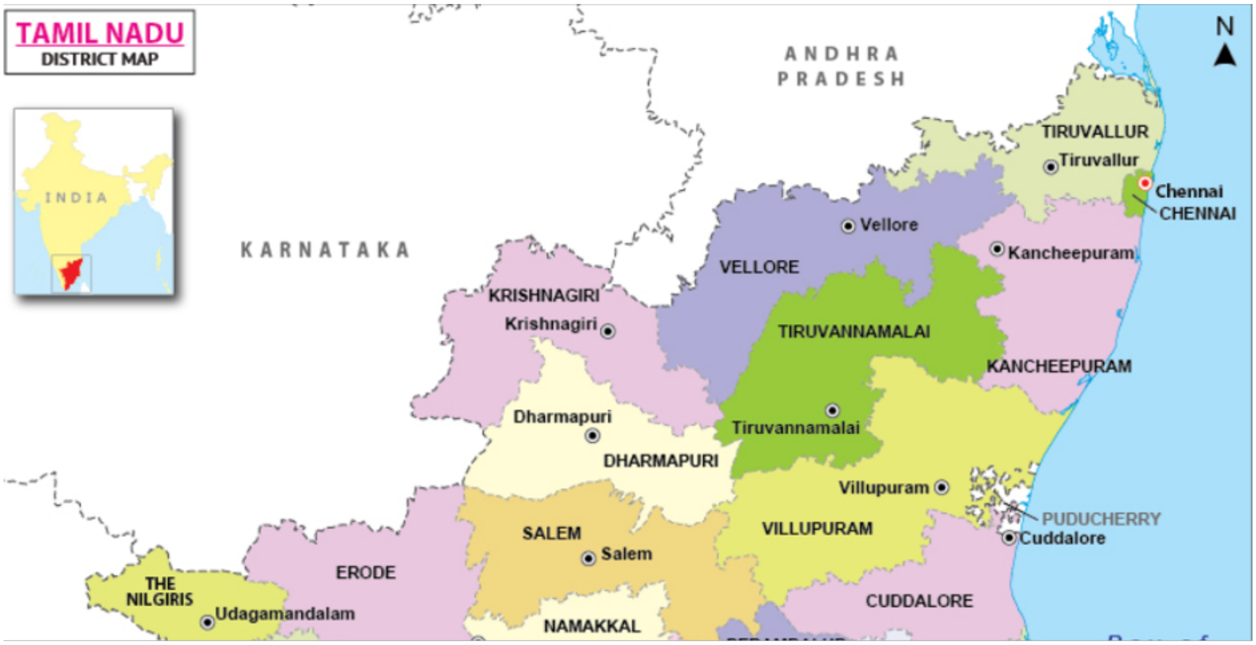

Source: https://www.mapsofindia.com/maps/tamilnadu/tamilnadu-district.htm. We refer to the Kanchipuram district based on the boundaries at the time we selected our candidate schools (mid-2019). As we were rolling out our intervention in November 2019, the Kanchipuram district split into two. Thus, some of our study schools are within what, since late 2019, is called the Chengalpattu district.

A.2 Tables

Table A1: Presence of soap: traditional soap vs. soapy bottle

	Baseline		Midline		Endline	
	Control	Treated	Control	Treated	Control	Treated
Avg presence of any soap	31.9%	26%	58.2%	90%	56.3%	87%
Avg presence of soapy bottle	20.1%	15.9%	51.7%	85%	40.1%	86.4%
Avg presence of traditional soap	17.4%	13.8%	28.2%	50.8%	23.8%	6.1%
Number of schools	92	82	100	100	79	77

Notes: Calculations are based off of the classroom observation sample. Soap or soapy bottle is considered present if it is both present and appears to be in use. Note that avg presence of soapy bottle + avg presence of soap $\neq$ avg presence of any soap since schools often had both soap and a soapy bottle present in the same classroom.

A.3 Analysis: subset samples

We repeat our analysis using samples consisting of only the schools that appear at baseline, midline, and endline in each of the classroom observation and rapid observation datasets.

Balance Tests

We begin by conducting balance tests to check whether the control and treatment groups in each of our resulting samples are balanced on covariates. Appendix Tables A2 and A3 compare observable school characteristics between the control and treatment groups for the samples from classroom observation data and rapid observation data, respectively. Our rapid observation sample appears to be balanced across a set of school characteristics and any differences between the control and treatment groups are not statistically significant. For our classroom observation sample, while individual t-tests indicate balance across various observables, we are able to reject a joint test of equality at the 90% significance level. Given this, the results for our presence of soap outcome variable using this smaller sample should be taken with a grain of salt.

Table A2: Balance Test: Classroom Observation Sample

	Control	Treated	Diff	p-value
Total num students	204.4	188.4	16.0	0.52
Total num classrooms	8.0	7.6	0.4	0.50
Num students in grades 3-5	101.7	106.8	-5.1	0.75
Percent female in grades 3-5	49.8	51.6	-1.8	0.12
Share Kanchipuram	0.6	0.5	0.1	0.27
Num handwash taps	8.1	7.5	0.6	0.49
Observations	71	60		

Notes: This table presents baseline characteristics of the schools in the treatment and control groups for the classroom observation sample. The individual p-values indicate that the two groups are balanced on various observables. A joint test of significance (via seemingly unrelated regression) across all observables is able to reject the null hypothesis that the two groups are balanced at a 90% significance level (P = 0.07).

Table A3: Balance Test: Rapid Observation Sample

	Control	Treated	Diff	p-value
Total num students	197.9	177.9	20.0	0.42
Total num classrooms	8.0	7.1	0.9	0.26
Num students in grades 3-5	96.5	97.2	-0.7	0.97
Percent female in grades 3-5	49.9	52.1	-2.3	0.14
Share Kanchipuram	0.6	0.4	0.2	0.09
Num handwash taps	8.2	7.6	0.6	0.63
Observations	48	37		

Notes: This table presents baseline characteristics of the schools in the treatment and control groups for the rapid observation sample. The individual p-values indicate that the two groups are balanced on various observables. A joint test of significance (via seemingly unrelated regression) across all observables also fails to reject the null hypothesis that the two groups are balanced (P = 0.21).

Presence of soap

At baseline, the average control school had soap and/or soapy bottles in 27.2% of classrooms and the average treatment school had soap in 25.0% of classrooms. This difference is not statistically significant.

As presented in Appendix Table A4, the share of soap in classrooms more than tripled to 85% for the average treatment school at midline (P < 0.01). The share also rose to 59% for the average control school at midline (P < 0.01). Once again, the rise in the share of present soap in control schools is likely due to teachers at treatment schools talking to peers at control schools or BRTEs not adhering to randomization. Finally, results at midline appear to have persisted at endline, with the average treatment school having soap in 85% of classrooms and the average control school having soap in 52.8% of classrooms (P < 0.01).

With this smaller sample, the midline double difference is given by 34.4 (P < 0.01) and the endline double difference is given by 28.8% (P < 0.01).

Table A4: Presence of soap and/or soapy bottles– by intervention arm and treatment status

	Baseline			Midline			Endline		
	Control	Treated	Diff	Control	Treated	Diff	Control	Treated	Diff
Avg presence of soap	27.2%	25%	-2.2	58.5%	85%	26.5***	52.8%	85%	32.2***
Avg num classrooms	2.7	2.8		2.6	2.6		2.7	2.8	
Number of schools	71	60		71	60		71	60	

Notes: Based on classroom observation sample. An individual school's observed presence of soap variable is calculated by dividing the number of observed classrooms with soap/soapy bottle present and in-use at that school by the total number of observed classrooms at that school (up to 3 classrooms). P-values were obtained via a one-sided t-test.
***p < 0.01, **p < 0.05, *p < 0.1

Handwashing before lunch

For treatment schools at midline, the share of schools with majority of students washing their hands with soap doubled from 27.0% to 56.8% (P < 0.01). Control schools in our sample saw slight decline, from 25% to 20.8% (see Appendix Table A5).

At endline, the shares converged slightly to 54.1% for treatment schools and 37.5% for controls. Furthermore, as we can see, the share of control schools where most/all students were observed handwashing before lunch increased back to near baseline value at endline, from the drop at midline (P < 0.05).

With this smaller sample, the midline double difference is given by 33.9 (P < 0.05) and the endline double difference is given by 14.5, though the endline double difference is not statistically significant.

Table A5: Handwashing before lunch– by intervention arm and treatment status

	Baseline			Midline			Endline		
	Control	Treated	Diff	Control	Treated	Diff	Control	Treated	Diff
Percent of schools with most/all students handwashing before lunch	25%	27%	2	20.8%	56.8%	35.9***	37.5%	54.1%	16.6*
Number of schools	48	37		48	37		48	37	

Notes: Based on rapid observation sample. P-values were calculated via a t-test.
***p < 0.01, **p < 0.05, *p < 0.1
